# Evaluation of Novel Design of Feed Spacer for Spiral-Wound Membranes Through CFD Simulations and Experiments

**DOI:** 10.3390/membranes16040123

**Published:** 2026-03-31

**Authors:** Meng Wang, Youxin Li, Lu Bai, Robert Field, Dengyue Chen, Bing Wang, Jun Jie Wu

**Affiliations:** 1 College of Environmental Science and Engineering, Nankai University, Tianjin 300071, China; 2 Department of Engineering Science, University of Oxford, Oxford OX1 3PJ, UK; 3 School of Pharmaceutical Sciences, Xiamen University, Xiamen 361005, China; 4 College of Engineering and Physical Sciences, Aston University, Birmingham B4 7ET, UK

**Keywords:** feed spacer, computational fluid dynamics, discrete phase model, biofouling, specific energy cost

## Abstract

This study proposes an innovative spacer design for use in spiral-wound membrane filtration systems as a high-performance alternative to conventional woven spacers. By eliminating interwoven filaments, this structure fundamentally reshapes flow patterns while maintaining mechanical support. A novel aspect of this methodology is the inaugural application of coupled computational fluid dynamics (CFD) and the discrete phase model (DPM) for modeling microbial particle transport and deposition dynamics, which has been a critical gap in prior studies that focused solely on hydrodynamic analysis without addressing biocolloid dynamics. Numerical simulations demonstrated that the novel design reduces stagnant zones by a significant amount compared to standard woven spacers and achieves a greater velocity uniformity. For all eight configurations of the novel design, the DPM-derived microbial distribution maps revealed a reduction of circa 65% in particle colonization density on the spacer surface, and this reaches a 77% reduction for the optimal design. These measurements directly linking structural geometry to antifouling efficacy provide mechanistic insight unattainable through conventional velocity field analysis alone. Experimental validation using optical coherence tomography (OCT) revealed a 40% reduction in TOC deposition, while confocal laser scanning microscopy (CLSM) quantified a 54% decrease in biofilm viability through adenosine triphosphate (ATP) measurements. The incorporation of the optimal spacer in the plate-and-frame test module demonstrated that the lower degree of fouling caused both a 23% increase in permeation flux together with 76% lower energy consumption compared to the commercial design.

## 1. Introduction

The global water crisis is a significant environmental concern, driven by rapid urbanization and pollution [1,2]. Key threats include industrial discharge, sewage and wastewater, oil spills, plastic pollution, and urban development pressures. At the same time, stricter water quality regulations and rising water demands have intensified the problem [3,4]. To address freshwater scarcity, membrane-based water treatment technologies have advanced significantly [5]. Reverse osmosis (RO) and nanofiltration (NF) are broadly utilized technologies, particularly for seawater desalination, drinking water purification, wastewater recycling, and brackish water treatment [6,7,8]. These advanced membrane separation technologies are transformative solutions to the world’s major water challenges, offering unrivaled advantages in terms of operational efficiency, energy savings, and system reliability. Their technological advantages mainly involve high process efficiency, energy optimization, and system stability and reliability.

Spiral-wound membrane modules (SWM) mainly utilize reverse osmosis (RO) and nanofiltration (NF) configurations, given their established efficacy in diverse water filtration applications [9]. SWM modules are widely used in water filtration, and they have the advantages of a small footprint and a large surface area [10]. The construction of SWM assemblies necessitates the placement of spacers between membrane layers and creating defined flow channels for the feed [11]. Together with the permeate spacer, the feed spacer is a vital element of the sandwich design. The feed spacers are designed to create turbulence, uniform flow distribution, and mitigate the deleterious effects of concentration polarization arising from filtration [12,13].

Previous research has established that hydrodynamic phenomena in membrane flow channels are critically governed by feed spacer geometry [12], and the geometric characteristics of conventional feed spacers—specifically flow angle, shape, filament spacing, and filament diameter—have increasingly become a focus of research [14,15,16,17]. Ahmad et al. demonstrated that cylindrical spacers exhibit superior suitability as feed spacer materials for spiral-wound membrane assemblies (SWMAs) within the ideal operating Reynolds number range (Re < 400), based on a comparative study of three distinct spacer geometries [18]. Huang et al. revealed that filament diameter variations critically govern hydraulic performance, observing that monolayered, non-uniform structures exhibit elevated flow resistance and compromised antifouling capacity [19]. Meysam et al. [20] evaluated five triply periodic minimal surface (TPMS)-based geometries in three cycles as feed spacers for direct contact membrane distillation (DCMD), and the results demonstrated that the use of TPMS spacers improves both heat and mass transfer efficiency in the system. Wang et al. [21] showed that their CFD- and RSM-optimized feed spacer (OPT-FS) outperformed the standard spacer (STD-FS), with about 5.5% less flux decline, 18–21% lower energy use, and 10–30% less surface fouling. Amin et al. [22] found that a novel double-thread spacer design, particularly with circular threads, is well-suited for achieving high-efficiency and low-energy performance in RO membrane elements.

Despite advancements in feed spacer geometry optimization, membrane biofouling remains a significant challenge. Research indicates that biofouling typically initiates on the feed spacer and gradually spreads to the membrane, resulting in overall performance decline [23,24]. Kerdi et al. found that perforated spacers can form microjets that generate enough unsteady turbulence to clean the membrane surface and reduce biofouling [25]. Huang et al. revealed that in feed spacer channels with varying inter-diaphragm gaps, biofouling exhibits distinct temporal progression patterns: shear stress prevails during initial fouling stages, while blockage effects dominate mid-to-late phase development. Their analysis indicated that targeted geometric modifications at critical fouling sites should be part of future research strategies [26]. It has also been known for many years that the modification of membrane surface properties—specifically charge distribution, surface roughness, and hydrophilicity—significantly reduces microbial attachment [12]. Yang et al. have combined structural optimization and surface modification to comprehensively inhibit biofouling and improve the permeability of membrane modules [27]. Kitano et al. fabricated antifouling-enhanced feed spacers by combining polypropylene (PP) and carbon nanotubes (CNTs), surpassing the performance of standard PP spacers [28]. Venkidusamy et al. mitigated membrane performance degradation by developing a multifunctional PDA-SP-cTA coating, demonstrating that in situ application suppressed biomass accumulation and that the increase in FCP was significantly smaller [29].

Through computational fluid dynamics simulations, Konda et al. optimized the feed channel design and found that by decreasing its cross-sectional area, widening the flow pathways around the fibers, and adjusting filament spacing, there was simultaneously lower FCP and improved mitigation of concentration polarization. Notably, their findings revealed that arranging fibers in X- or V-shaped configurations further enhances performance by minimizing surface fouling and reducing biofouling and scaling risks [30]. Despite thorough investigations into the feed spacer of geometry effects on mass transfer and FCP drop, their precise relationship with biofouling has received little attention [31,32]. Brazhenko et al. [33] investigated the purification of AMG-10 hydraulic oil from smokeless coal particles using a rotating perforated cylinder through experiments and simulations. Their analysis of the feed-side flow field revealed that primary and secondary vortices influence the subsequent cleaning efficiency of the filter. Therefore, further study of the feed spacer design is crucial for addressing biofouling challenges and improving module performance, which are both key to the important metric of specific energy consumption and to module cleaning and its associated longevity.

One of the key tools to aid the above studies is computational fluid dynamics, which facilitates quantitative characterization of hydrodynamic phenomena, thus establishing critical linkages between feed spacer geometry and filtration process efficiency [12]. Momeni et al. demonstrated via CFD simulations that fiber density significantly influenced infiltration flux, with the feed flow rate exerting a substantially greater impact on permeate flux enhancement than permeate-side conditions [34]. Ronen et al. characterized the spatial dispersion of silver ions across both the feed channel and membrane interfacial region by using numerical simulation [35]. Today, another tool is available, namely, 3D printing. Rapid advances now enable speedy, cost-effective fabrication of complex spacer geometries. This synergy offers significant potential for developing novel feed spacers to experimentally evaluate geometric impacts [10,36,37,38,39].

Our present study introduces a novel spacer design to improve module performance and reduce energy consumption compared to traditional commercial spacers. By combining three facets—(i) computational fluid dynamics (CFD) simulations together with (ii) discrete phase model (DPM) and (iii) biofouling experiments—the effects of various aspects of the design of the novel spacer (particularly filament distance, hydrodynamic angle, and surface curvature) on hydrodynamic performance, filtration efficiency, and fouling behavior were systematically investigated. Eight feed spacer configurations with varying geometric parameters were designed and analyzed to evaluate their filtration and biofouling performance. The CFD simulations revealed how the geometric characteristics of the feed spacers influence hydrodynamic behavior and biofouling development within the channel. The extensive study enabled rigorous quantification of spacer design–module performance correlations, giving mechanistic insights that will facilitate the future development of energy-efficient filtration systems.

## 2. Materials and Methods

### 2.1. Feed Spacer Geometry

**Figure 1:**
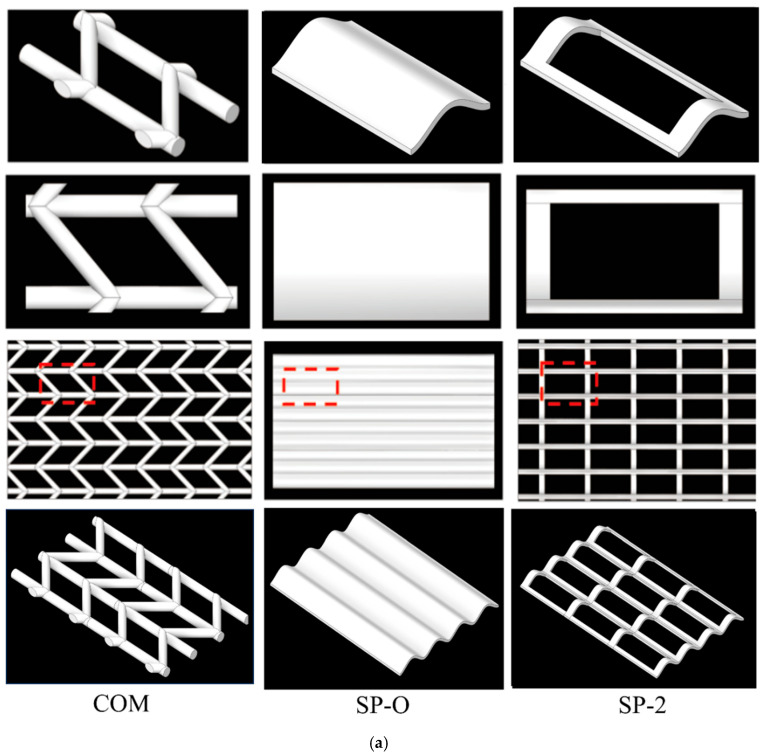

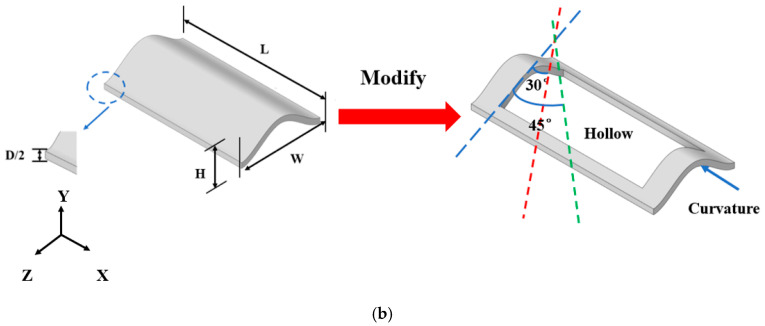
(**a**) Schematic and photographic views of commercial and SP-type spacers from multiple angles. (**b**) SP-X series dimension diagram.

Figure 1a depicts the commercial spacer, the SP-O spacer, and one of the SP types; they share identical 1.2 mm node heights. Whereas the commercial spacer featured 1 mm filament diameters, both SP-O and SP-2 variants employed 0.5 mm diameters. The vertex supports of SP-O and SP-2 are 1 mm × 0.7 mm rectangles. SP-2 is further modified by hollowing out a 3.1 mm × 7 mm rectangular section from the SP-O structure. The SP-X series, derived from the SP-O base design, includes variations in filament distance (SP-1 to SP-3), hydrodynamic angle (SP-4 and SP-5), and surface curvature (SP-6 and SP-7). Detailed dimensions for all configurations are provided in Table 1 and Figure 1b. The angle θ is defined with respect to the positive direction of the *Z*-axis (i.e., the light-blue dashed line) as the reference and is positive for a counterclockwise direction. It refers to the angle between the tangent to the centerline of the spacer filament at any given point and the *Z*-axis. Curvature (κ): this is a mathematical measure of the local bending degree of the spacer filament centerline, defined as the reciprocal of the radius of the osculating circle (κ = 1/R). For instance, a curvature value of $\frac{1}{0.5}$ corresponds to a radius of curvature of 2 mm.

Eight distinct feed spacer geometries were digitally modeled with the commercial software Space Claim 2023 R1 for subsequent fabrication via 3D printing. The 3D printer, ProJet 3510HD Plus (Rock Hill, SC, USA), provides a print accuracy of 0.016 mm, and the material used in the printing was VisiJet M3 Crystal, a translucent, hard material that is heat-resistant and stable.

### 2.2. CFD Simulation

#### 2.2.1. Mesh Independence and Computational Conditions

The eight distinct feed spacer geometries modeled in SpaceClaim were individually imported into ANSYS Fluent 2023 R1 for computational fluid dynamics (CFD) analysis.

**Figure 2:**
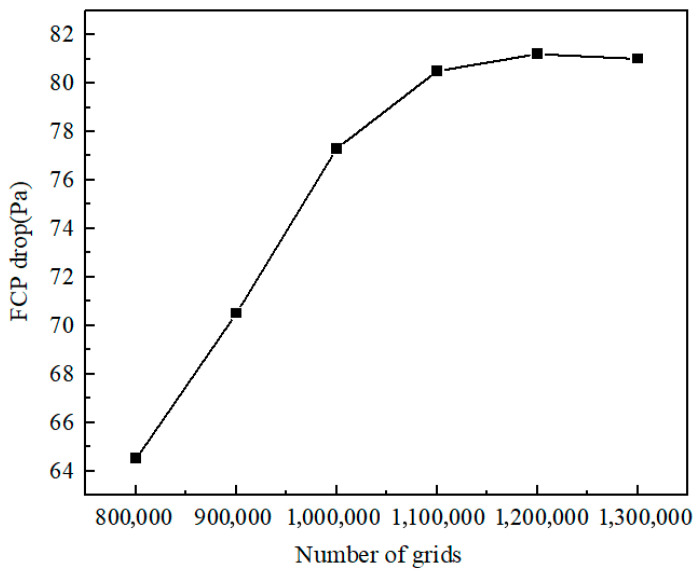
Effect of grid number on the simulated FCP drop.

Due to the relatively complex internal flow channel structure of the spiral-wound membrane module, the entire computational domain is discretized using unstructured hexahedral elements. As shown in Figure 2, it is evident that when the number of grids exceeds 1.2 million, the impact on the FCP drop is minimal. The computational domains were discretized using meshes ranging from approximately 1.28 million to 1.57 million elements per channel configuration. A mesh independence study was conducted, confirming that the resulting computational errors in the CFD solutions remained below 5%.

In this study, the feed inlet was assumed to be an incompressible fluid at a set velocity. The permeate outlet pressure was set to atmospheric pressure and defined as a pressure outlet. Given that the permeation velocity through the membrane is significantly lower than the fluid flow velocity in the channel and thus does not affect the internal flow, the bottom surface of the channel was assumed to be impermeable. No-slip smooth conditions were applied at both channel walls, and the surfaces of the feed spacer were defined as wall boundaries.

#### 2.2.2. Numerical Method and Governing Equation

A representation of the fluid flow phenomena requires two conservation laws—conservation of mass and conservation of momentum—assuming incompressible Newtonian fluids and no energy exchange. The Reynolds number (defined using feed channel height and superficial velocity) within the feed spacing channel generally remains below 200 [40]. According to the Reynolds number formula, it is calculated that the Reynolds numbers for both the commercial and SP series are less than 200, thus meeting the conditions for laminar flow. Empirical studies indicate that commercial spiral-wound membrane systems operate with characteristic flow velocities between 0.07 and 0.15 m per second [41]. Therefore, the inlet velocity was set as 0.12 m per second, with the outlet pressure at atmospheric pressure. The convergence criterion for the calculation is 10^−5^. The computational model treated the working fluid as an incompressible, steady-state laminar flow of pure water under standard conditions (25 °C, *ρ* = 997 kg/m^3^, *μ* = 8.899 × 10^−4^ Pa·s). Accordingly, the continuity equation (Equation (1)) and momentum equations (Equations (2)–(4)) were adopted.

(1) $$\frac{\partial u}{\partial x}+\frac{\partial \nu }{\partial y}+\frac{\partial w}{\partial z}=0$$

(2) $$u\frac{\partial u}{\partial x}+v\frac{\partial u}{\partial y}+w\frac{\partial u}{\partial z}=-\frac{1}{\rho }\frac{\partial P}{\partial x}+\frac{\mu }{\rho }\left[\frac{\partial^{2}u}{\partial x^{2}}+\frac{\partial^{2}u}{\partial y^{2}}+\frac{\partial^{2}u}{\partial z^{2}}\right]$$

(3) $$u\frac{\partial v}{\partial x}+v\frac{\partial v}{\partial y}+w\frac{\partial v}{\partial z}=-\frac{1}{\rho }\frac{\partial P}{\partial y}+\frac{\mu }{\rho }\left[\frac{\partial^{2}\nu }{\partial x^{2}}+\frac{\partial^{2}\nu }{\partial y^{2}}+\frac{\partial^{2}\nu }{\partial z^{2}}\right]$$

(4) $$u\frac{\partial w}{\partial x}+v\frac{\partial w}{\partial y}+w\frac{\partial w}{\partial z}=-\frac{1}{\rho }\frac{\partial P}{\partial z}+\frac{\mu }{\rho }\left[\frac{\partial^{2}w}{\partial x^{2}}+\frac{\partial^{2}w}{\partial y^{2}}+\frac{\partial^{2}w}{\partial z^{2}}\right]$$

Here, u, v, and w denote the velocity components along the x, y, and z coordinate system (m/s), while *ρ* is the liquid density (kg/m^3^), *μ* is the dynamic viscosity, and P is the pressure (Pa).

In order to better study biofouling formation, the discrete phase model (DPM) in Fluent 2023 R1 software is employed for simulation using the Euler–Lagrange method, where the fluid phase as a continuous medium is solved by solving the Navier–Stokes equations, while the particle phase is solved by tracking the trajectories of a large number of particles, bubbles, or droplets. Therefore, the discrete phase model will be used in the future to qualitatively assess biofouling formation in different compartments at different constant fluxes. Herein, we use it as a foundation to assess global trends, and it will be a guide for future experiments. When the volume fraction occupied by the particle phase in the flow field region is small enough, the particle–particle interaction can be neglected. Hence,

(5) $$m_{p}\frac{d{\vec{u}}_{p}}{dt}=m_{p}\frac{\vec{u}-{\vec{u}}_{p}}{\tau_{r}}+m_{p}\frac{\vec{g}\left(\rho_{p}-\rho \right)}{\rho_{p}}+\vec{F}$$

where $m_{p}$ is the particle mass, $\vec{u}$ is the fluid phase velocity, ${\vec{u}}_{p}$ is the particle velocity, ρ is the density of the particle, $\vec{F}$ is an additional force, $m_{p}\frac{\vec{u}-{\vec{u}}_{p}}{\tau_{r}}$ is the drag force, and $\tau_{r}$ is the droplet of particle relaxation time [42] calculated by

(6) $$\tau_{r}=\frac{\rho_{p}d_{p}^{2}}{18\mu }\frac{24}{C_{d}Re}$$

where μ is the molecular viscosity of the fluid, $d_{p}$ is the particle diameter, and *C_d_* is the drag coefficient [43], which is defined as

(7) $$C_{d}=\left\{\begin{array}{l} \frac{24}{Re}\left(1+0.15{Re}_{p}^{0.687}\right)\;\;\;Re<1000\\ 0.44\;\;\;\;\;\;\;\;\;\;\;\;\;\;Re>1000 \end{array}\right.$$

*Re* is the relative Reynolds number, which is defined as

(8) $$Re\equiv \frac{\rho d_{p}\left|{\vec{u}}_{p}-\vec{u}\right|}{\mu }$$

### 2.3. Hydraulic and Biofouling Experiments

#### 2.3.1. Experimental Setup

In this study, hydraulic performance, filtration performance, and fouling performance were tested on feed spacers in a filtration system. The experimental setup comprised three parallel modules, each equipped with distinct feed spacers. Nanofiltration membranes (NF, rejection rate ≥ 99.0%, Zhongke Ruiyang, Beijing, China) were employed due to their prevalent use in pretreatment processes for desalination and wastewater treatment and propensity to foul. The experiments were conducted as short-term parallel tests over five days.

**Figure 3:**
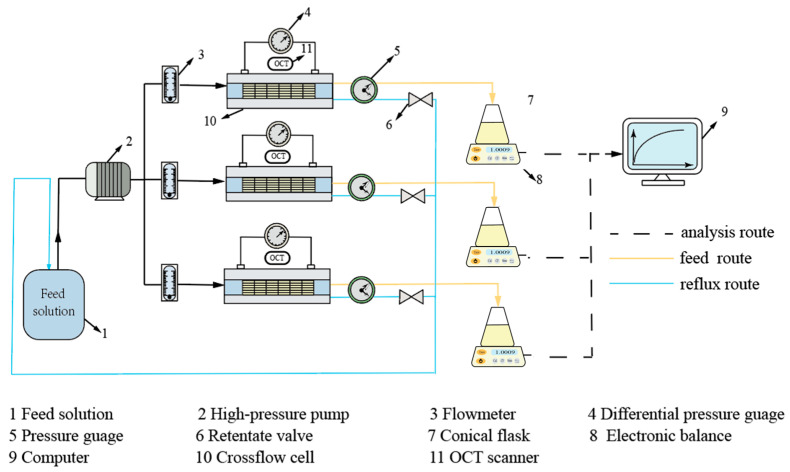
Schematic diagram of experimental rig.

During the hydraulic performance experiment stage, a 50 L feed tank delivered the feed solution via a booster pump (CHLP-RP, NURET, Milan, Italy) to three parallel modular pathways. Each pathway included a flow meter, a cross-flow cell (66 × 40 × 1.2 mm^3^), a retentate valve, an electronic balance, and a conical flask for collecting the final permeate. Monitoring of the feed channel pressure drop across each module was accomplished through a differential pressure transmitter. The detailed experimental configuration is illustrated in Figure 3. The experimental flow cell was equipped with an OCT-compatible viewing port (Optoprobe OPIMG, Oxfordshire region, UK) for dynamic fouling layer characterization. The feed tank was replenished with water and nutrients twice daily to maintain consistent experimental conditions.

#### 2.3.2. Operation Conditions

During the experimental preparation phase, the membrane was immersed in deionized (DI) water for 24 h to remove surface-bound contaminants and particulates. After assembly of the module, the membrane was subjected to hydraulic compaction in the cross-flow configuration, utilizing distilled water at 0.48 MPa transmembrane pressure. The results indicate that the initial fluxes of the SP-O, SP-2, and Com systems were 29.4 LMH, 26.5 LMH, and 23.2 LMH, respectively. Pressurization was maintained until permeate production reached a steady state. To align with the boundary conditions of the CFD simulation, the inlet flow volume of each cross-flow cell was adjusted to 200 mL/min, with different feed spacers inserted into the three cells. Both FCP and permeate fluxes were observed for different feed spacers, repeated three times, and averaged. During the 5-day biofouling experimental runs, where the target organisms were opportunistic bacteria in tap water, synthetic nutrient solutions were used to accelerate biofouling formation by supplying the necessary nutrients. These were added per day throughout the whole of the experimental run. The material composition of the nutrient solution was CH_3_COONa, NaNO_3_, NaH_2_PO_4_, CaCl_2_⋅2H_2_O, and MgCl_2_⋅2H_2_O. The concentration of the nutrient solution was controlled at 0.5 mg/L Mg^2+^, 0.5 mg/L Ca^2+^, and 2 mg/L C with a C: N: P mass ratio of 100:20:10. The feed solution was adjusted to pH 10 using sodium hydroxide to create an inhospitable environment for bacterial colonization within the dosing container [44].

#### 2.3.3. Biomass Analysis

Biofouling characterization was conducted using a comprehensive multi-technique approach to assess both the organic and biological components of the accumulated deposits. Total Organic Carbon (TOC) analysis was employed to quantify the overall organic content and evaluate the extent of carbon-based fouling. Adenosine triphosphate (ATP) quantification was performed as a rapid and sensitive method to determine viable microbial biomass within the biofouling layers. Additionally, confocal laser scanning microscopy (CLSM) was utilized to provide high-resolution, three-dimensional visualization of the biofilm architecture, enabling a detailed examination of the microbial community structure.

Representative membrane and feed spacer samples (1 × 2 cm^2^) underwent sequential chemical extraction for TOC analysis. Specimens were first immersed in 10 mL 0.2% HCl solution and agitated at 150 rpm (25 °C, 24 h), followed by alkaline treatment with 10 mL 0.26% NaOH solution under identical conditions. For TOC determination, the combined samples were sequentially filtered through 0.45 μm microfilters and analyzed using a multi N/C 3100 system (Analytik Jena, Thuringia, Germany). Biofilm accumulation on the membranes was quantified via ATP analysis. For measurement, 1 × 1 cm^2^ sections were excised from membrane centers, with ATP content determined using the luciferin–luciferase assay [45]. Biomass samples were homogenized in 1.5 mL sterile saline solution via vortex mixing for 3 min in centrifuge tubes. To avoid errors, the middle part of the membrane was selected as the most homogeneous part of the biofouling, and 1 × 2 cm^2^ samples from this region of the membrane were randomly chosen as the CLSM test section. The 1 × 2 cm^2^ samples were taken and stained with the LIVE/DEAD BacLight Bacterial Viability Kit for 15 min under light-avoidant conditions at room temperature. The data were subsequently captured using a Confocal Laser Scanning Microscope (CLSM, LSM880 with Airyscan, Zeiss, Thuringia, Germany) at a laser wavelength of 488 nm.

## 3. Results and Discussion

Before reporting and discussing the filtration performance with the selected spacers in Section 3.4, the next three sections assess the insights gained from experimental and CFD evaluation of the hydraulic performance of the selected spacers (Section 3.1), the insights from the use of the discrete phase model (Section 3.2), and the insights from the various measurements regarding the biofilms on the three spacers (Section 3.3).

### 3.1. Hydraulic Characterization of Feed Spacers

#### 3.1.1. FCP Drop

**Figure 4:**
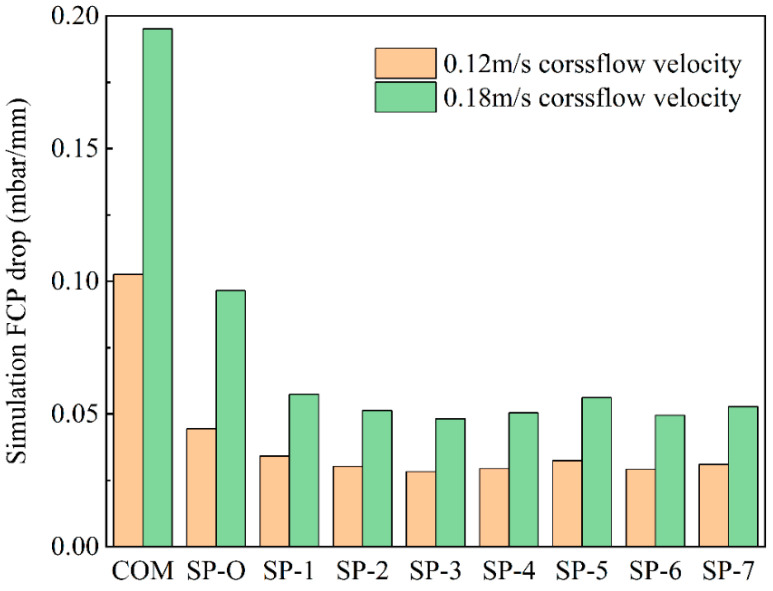
Evaluation of pressure drop for the nine feed spacer models at two cross-flow velocities. The fluid was pure water.

The FCP pressure drop was used as a measure to characterize the hydraulic performance of the various feed spacers. Figure 4 indicates that at both low and high crossflow velocities, the commercial woven feed spacer has the greatest pressure drop, with values being about double that of the SP-O type and around three times that of the SP series spacers. This indicates that the SP feed spacers, in particular, have the potential to reduce energy consumption compared with commercial feed spacers. Increased filament spacing in SP-1/2/3 feed spacers effectively lowered the FCP drop. The SP-2/4/5 feed spacers altered the hydrodynamic angle and did not progressively increase the pressure drop as the hydrodynamic angle was increased from 0° to 30°. Presumably, the openness of the feed spacer due to the hollow feature shown in Figure 1b mitigates any changes in flow paths. The effect of angle-induced changes on pressure drop may not be uniform across feed spacers, as Figure 4 clearly shows that beyond 30°, the pressure drop increases gradually with an increasing angle. This SP-2/6/7 feed spacer changes the curvature, which is subtle compared to the original SP-2. Different spacers can be selected for different situations; the SP-7 with high curvature is more appropriate at lower flow rates whilst the SP-6 with low curvature is more appropriate at higher flow rates. A deeper study of the effect of curvature on spacer design will be pursued in future studies.

Based on the simulation results, COM, SP-O, and SP-2 feed spacers were chosen for experimentation. Under the corresponding cross-flow velocity experimental conditions, hydraulic performance and biofouling experiments were carried out with these three feed spacers in parallel experiments.

**Figure 5:**
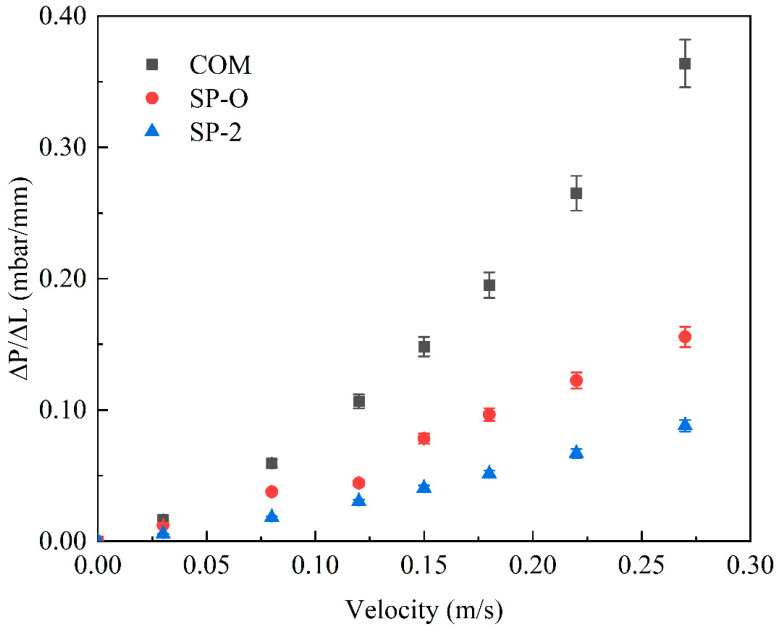
Pressure gradient as a function of crossflow velocity for the three spacers, COM, SP-O, and SP-2.

Figure 5 indicates the pressure drop across the system (FCP) of three types of feed spacers as a function of cross-flow velocity. As the fluid velocity increases, the FCP of the commercial spacer follows a typical nonlinear relationship, but the novel spacer displays a near linear relationship. So, whilst for the commercial spacer the pressure drop escalates more rapidly under high flow conditions, where viscous dissipation and kinetic energy losses become more pronounced, this change in gradient is far less pronounced for SP-O and SP-2 compared with COM.

**Figure 6:**
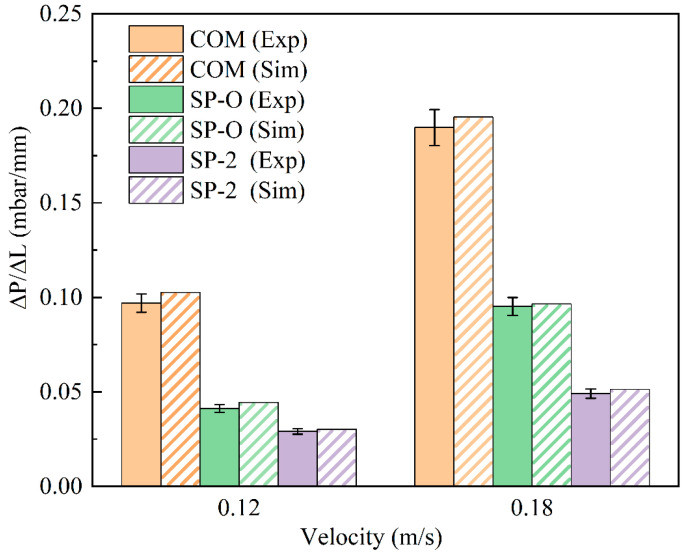
Comparison of simulation results with experimental measurements of two flow rates for spacers COM, SP-O, and SP-2.

As shown in Figure 6, for both 0.12 m/s and 0.18 m/s, the CFD simulation results were in good agreement with the experimental results; the difference was less than 5%. This indicates that the chosen CFD tools can be used to evaluate the FCP of similar spacers in the future. Figure 6 shows that the SP-2 spacer with the hollow feature is superior to the other two at both low and high flow rates, indicating that the inclusion of the hollow feature generates advantages.

#### 3.1.2. Velocity and Shear Stress

In the CFD simulations, the upper and lower walls of the model are symmetrical with the same model profile. The monitoring point at 0.12 mm (one-tenth of the channel height) is strategically selected to validate simulation accuracy. This location is within the critical near-wall region, where velocity and concentration gradients are most pronounced. Therefore, only the bottom membrane wall is analyzed in the discussion.

**Figure 7:**
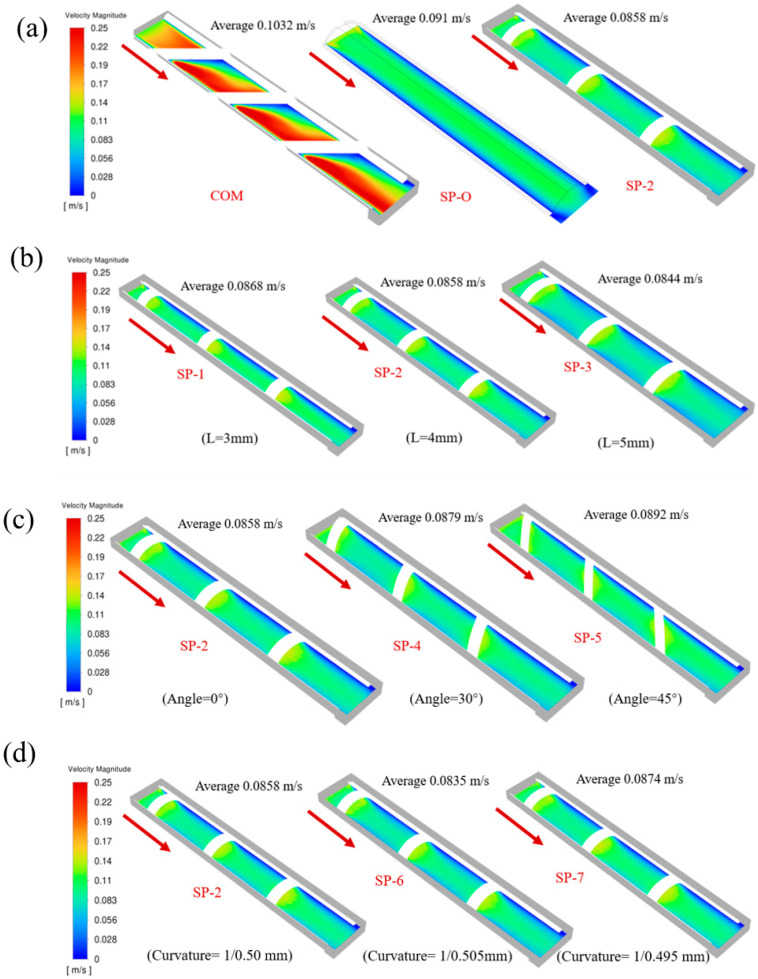
The flow velocity field near the lower membrane surface (0.12 mm distance) in the feed spacer channel for different parameters. (**a**) COM, SP-O, and SP-2, (**b**) filament distance, (**c**) mesh angle, and (**d**) surface curvature.

In the feed spacer configuration, the woven type generates more pronounced secondary flows and stable recirculation zones than the SP type. The SP-O and SP-2 are similarly constructed, with little difference in velocity. Nevertheless, localized flow separation and small-scale vortices are generated at some of the nodes of the SP-2 configuration (Figure 7a). The near-membrane horizontal velocities decrease with an increasing filament length (L) (from 0.0868 m/s for SP-1 to 0.0844 m/s for SP-3). This reduction is attributed to diminished flow disturbance and weaker induced secondary flows resulting from a lower filament distribution density along the flow path (Figure 7b). For the spacer mesh angle (Angle), (perpendicular to the feed stream), the distance between neighboring spacer grids can be reduced, which produces stronger flow deflection and more significant flow separation (Figure 7c). There may be some curvature in the spacer mesh itself, so the change in angle is not a significant change. In the vertical direction, when the curvature gradually increases, the velocity increases from 0.0835 m/s in SP-6 to 0.0874 m/s in SP-7. This is because the gap between the grid and the bottom membrane is larger at high curvature, which reduces flow constriction and mitigates the intensity of flow separation (Figure 7d).

**Figure 8:**
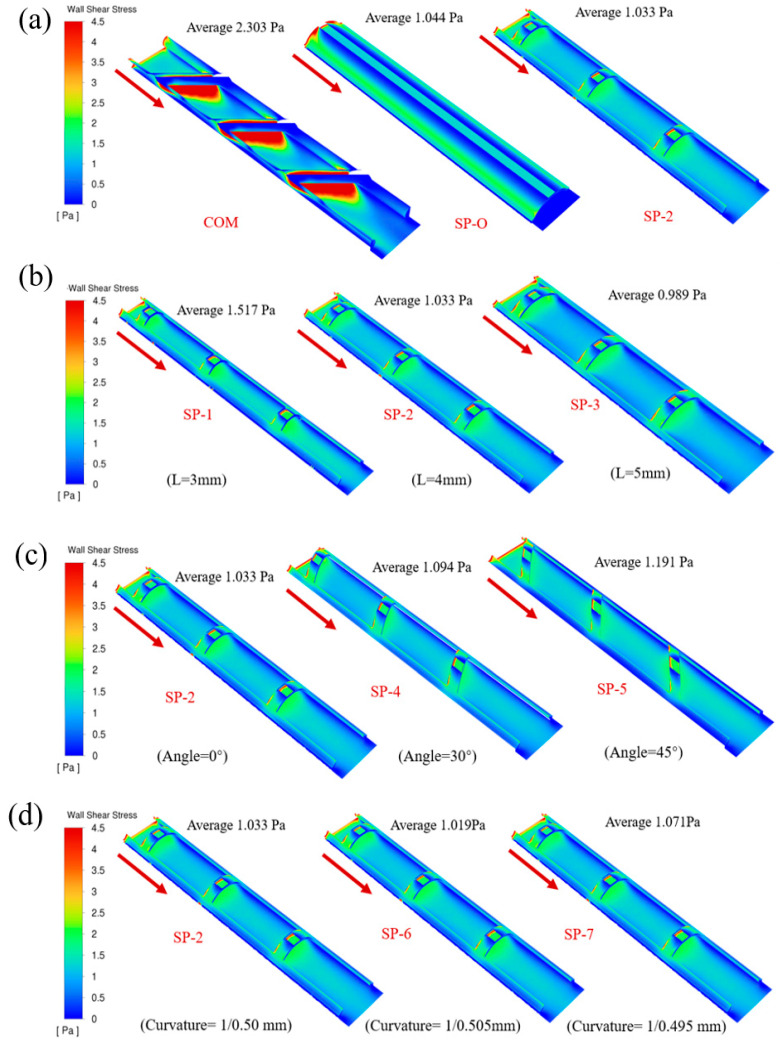
Shear stress distributions were measured on the membrane surfaces with different spacers in the feed channels. (**a**) COM, SP-O, and SP-2, (**b**) filament distance, (**c**) mesh angle, and (**d**) surface curvature.

Figure 8 clearly shows the characteristics of the shear stress distribution on the membrane wall at the bottom of the feed spacer. Through comparative analysis, it can be seen that the traditional woven commercial grids produce an obvious flow separation phenomenon when the fluid flows through them due to the periodic arrangement of the fiber structure, creating significant non-uniformity in the distribution of shear stresses. In contrast, the novel SP spacers are able to facilitate lateral mixing of the fluid, reduce the dead zones, and thereby enhance uniformity.

**Figure 9:**
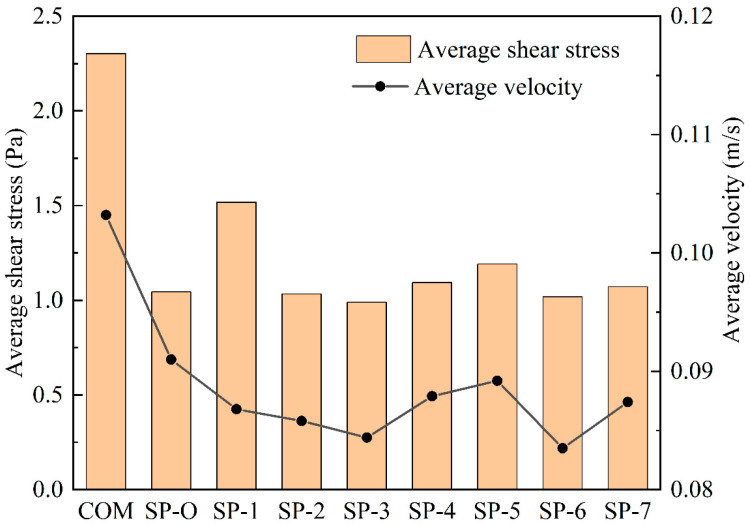
Comparison of the average velocity and average shear stress of different spacer surfaces at 0.12 mm from the bottom surface of the membrane.

Figure 9 provides a quantitative comparison of the average velocity and average shear stress for the different spacers at a position that is 0.12 mm from the bottom surface of the membrane. Notably, SP-3 exhibits the lowest values for both parameters, suggesting that increasing the filament distance during the interval effectively minimizes hydrodynamic forces at the membrane interface. This reduction in velocity and shear stress can be attributed to the modified flow dynamics induced by the wider filament spacing, which likely decreases flow constriction and mitigates turbulence near the membrane surface.

### 3.2. Particulate Concentration Distribution

Previous studies primarily focused on investigating the hydrodynamic changes induced by feed spacers within spiral-wound membrane modules, yet they rarely utilized simulation methods to predict pollutant distribution. Therefore, the discrete phase model (DPM) was employed. Although it does not model biofouling itself, the simulation results provide qualitative yet mechanistically insightful perspectives regarding accumulation trends and associated distribution patterns. Finally, further fouling experiments were conducted, enabling a more comprehensive understanding of the advantages and limitations of the novel spacer structure.

The steady-state discrete phase model (DPM) enhances simulation accuracy by modeling particle trajectories within continuous flow fields under well-characterized inlet and outlet boundary conditions. This has facilitated a qualitative analysis of the distribution of particulate concentration for the different feed spacers under comparable conditions. Since biofouling usually accumulates on the membrane surface, and the CFD simulation profiles of the two walls are essentially the same, the analysis is conducted on the bottom membrane wall.

**Figure 10:**
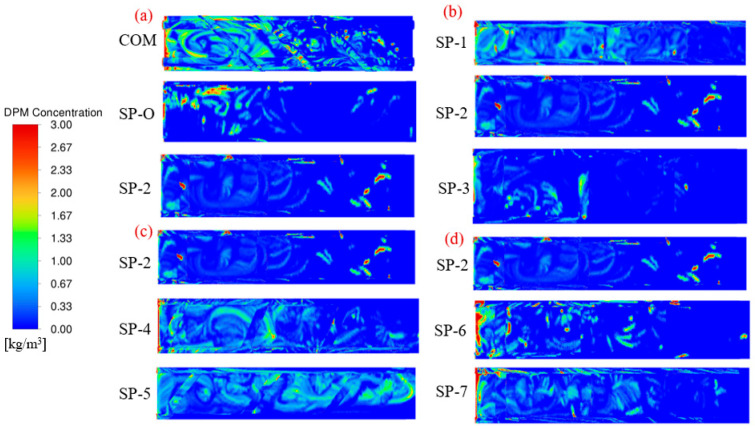
The distribution of particle concentration in different feed spacers. (**a**) COM, SP-O, and SP-2, (**b**) filament distance, (**c**) mesh angle, and (**d**) surface curvature.

The particle concentration with the woven feed spacer (COM) is higher than with the SP configurations; see Figure 10a. It can be observed that pollutants tend to accumulate more on the COM feed spacer. In contrast, the SP-O and SP-2 feed spacers show a significant reduction in the number of contaminated particles. This illustrates the distribution of particulate concentration for three different spacers, showing that both SP-O and SP-2 spacers are effective in reducing particulate matter accumulation.

Figure 10b illustrates the effect of increasing the feed spacer’s filament distance and confirms that SP-2 has desirably low concentrations of particulate matter. This demonstrates that a larger spacing helps to reduce pollutant accumulation, whereas a narrower gap between the spacer filaments and the membrane promotes bacterial deposition and growth. Figure 10c shows that an increase in angle will increase the particulate matter concentration, confirming that SP-2 is superior to SP-4 and SP-5. Increasing the angle (toward the feed flow) may reduce the distance between adjacent spacer nets, making it easier for contaminants to accumulate on the membrane surface and the nets. Figure 10d illustrates the influence of small changes in curvature and confirms the superiority of SP-2 over SP-6 and SP-7. The simulation results suggest that modest changes in surface curvature appear to have little effect on contaminant accumulation, which is probably because the flow state inherently aligns with the surface curvature.

Therefore, increasing the filament spacing reduces fouling primarily by widening flow channels, which lowers resistance and enhances local velocities and wall shear stress, thereby promoting particle removal. Conversely, a larger filament angle increases fouling risk, as inclined fibers disrupt the main flow, creating persistent low-velocity zones or vortices that trap contaminants. Notably, minor curvature adjustments have a negligible effect, highlighting that macroscopic parameters, such as spacing, angle, and overall geometry, take precedence over subtle shape refinements.

**Figure 11:**
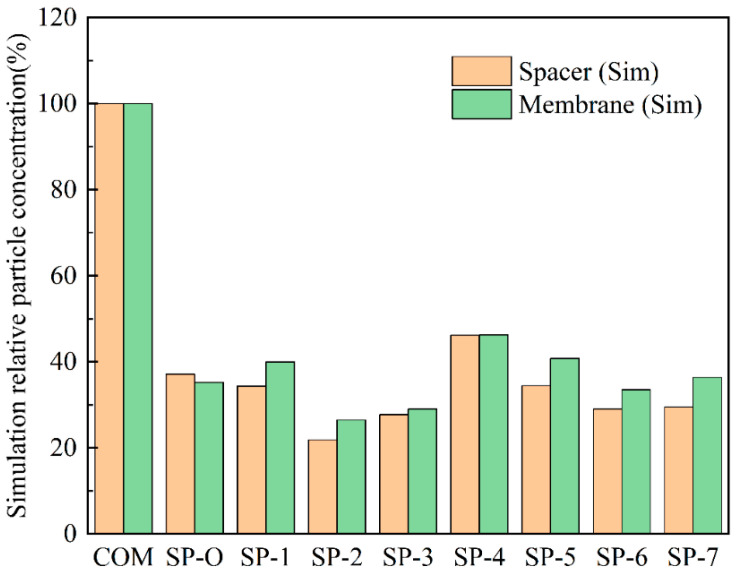
Particle concentration as given by the DPM model for different feed spacers. The values are relative to those on COM being set at 100%.

The superiority of SP-2 can be demonstrated in another way by setting the values for the particulate matter concentrations on COM at 100% and then comparing the values for each SP-X to COM. The results are given in Figure 11 for both the membrane surface and the spacers themselves. Although the discrete phase model (DPM) simulations are definitely not a modeling of biofouling per se, the results do offer a qualitative yet mechanistically insightful insight into the tendency to accumulate and the distribution of the accumulation.

### 3.3. Fouling Accumulation

**Figure 12:**
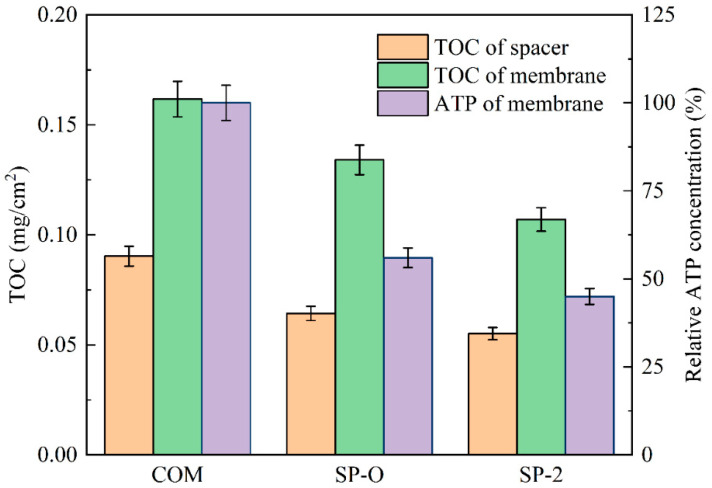
The TOC concentration and ATP concentration on the membrane surface and the spacer after experimental runs lasting 5 days.

Given that hydrodynamic conditions play a critical role in influencing biofouling behavior, it is essential to thoroughly investigate how the spatial geometry and structural design of spacers affect biofouling development. Figure 12 displays measures of fouling accumulation (TOC on spacer, TOC on membrane, and ATP on membrane) for three different feed spacers. It can be observed that SP-2 outperforms SP-O, which in turn outperforms COM. The middle part of the selected module was used for sampling to avoid errors. There is greater accumulation on the membrane surface compared to the spacers, and this is probably inherent in filtration, convecting material to the membrane surface where microbial material and many nutrients are rejected. Also, these spacers provide few attachment points, especially attachment points subject to low flow.

**Figure 13:**
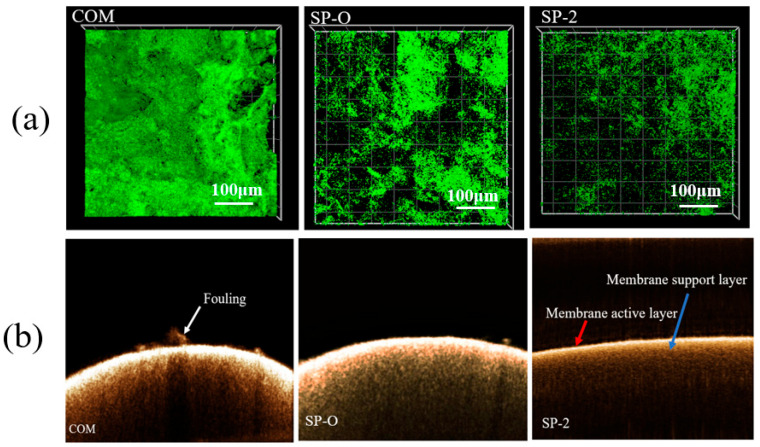
Images taken after 5-day operational period. (**a**) CLSM images of biomass-filled channels with different spacers, COM, SP-O, and SP-2. As noted in the image, scale bars are for 100 µm. (**b**) OCT-based fouling characterization of the membrane surface.

Figure 13a presents the CLSM images of the biomass in the channels fitted with different spacers. The green regions quantitatively indicate the accumulated biomass for the same feed flow rate. It is conspicuously observable from the figure that the quantity of biomass is ranked as follows: COM comes first, followed by SP-O, and finally SP-2. The biomass accumulation of SP-2 is sparser, suggesting that there is less hindrance from the spacer during the biofouling process. This implies that this spacer is superior to the others. To investigate the function of the spacer structure on steady-state fouling, two-dimensional OCT scans were performed. Figure 13b illustrates the OCT images obtained following the 5-day biofouling experimental runs. Based on the OCT results, it can be seen that SP-2 has a thinner biofouling layer, relative to COM and SP-O. Different colored arrows are used to indicate the active layer of the membrane (bright line in the center), the NF membrane (below the active layer), and the fouling layer (top of the active layer).

The DPM simulations in Section 3.2 are in accord with these findings. The DPM model indicates that the woven feed spacer (COM) favored particulate fouling, and the biofouling results also indicate that the biofilm is thicker and more concentrated in the module fitted with the COM feed spacer. Its filament structure clearly creates microenvironments that favor deposition, be it particulate or biomass. Furthermore, low-shear zones near filament intersections allow thicker biofilm development. The consistency between the DPM and experimental results underscores that spacer geometry optimization, rather than membrane modifications alone, should be prioritized for fouling mitigation in systems dominated by spacer-associated biofouling.

### 3.4. Filtration Performance

**Figure 14:**
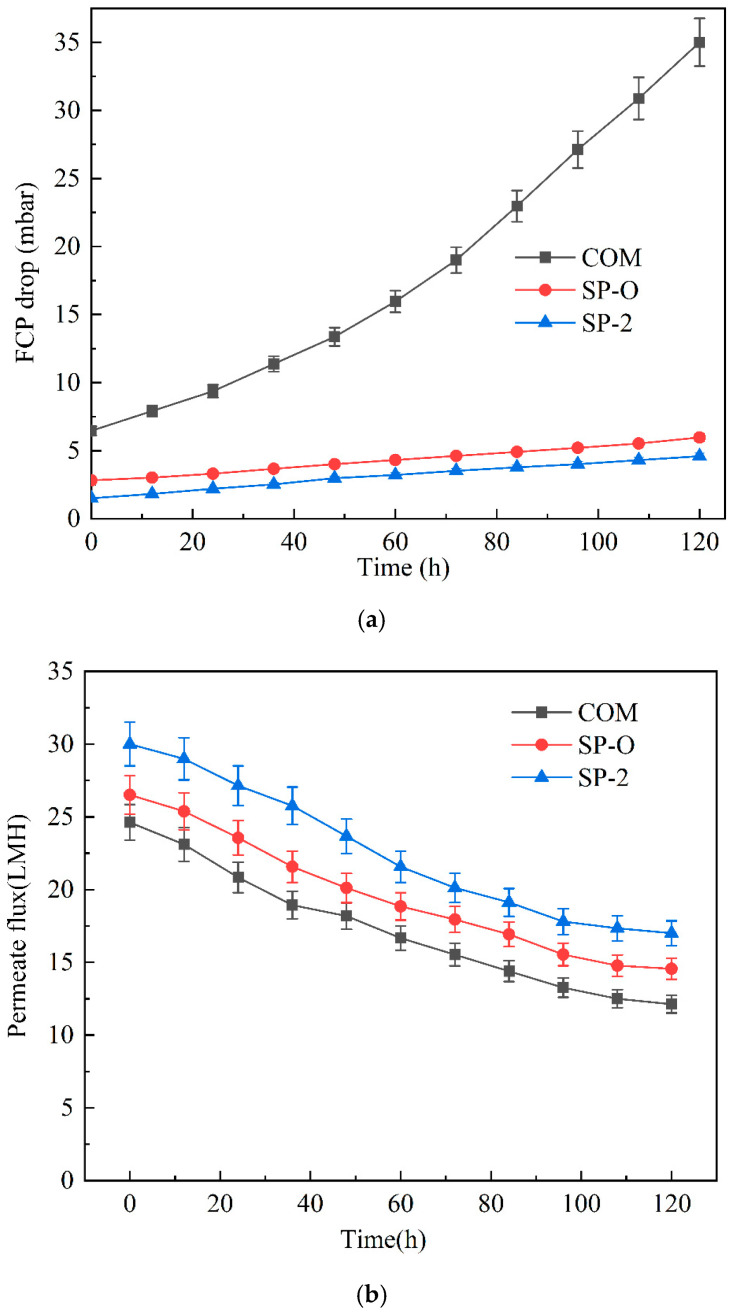
Long-term operation at a constant crossflow velocity of 0.12 m/s with 0.76 MPa transmembrane pressure. (**a**) Top: Evolution of the pressure drop behavior of the three spacers. (**b**) Bottom: Time evolution of volumetric flux.

As shown in Figure 14a, the three different configurations of spacers (COM, SP-O, and SP-2) exhibit a gradual increase in the pressure drop (FCP) with time during the 5-day experimental run, but there is a significant difference in the magnitude of the increase and the kinetic characteristics. With COM, FCP increased dramatically from 6.46 mbar to 35 mbar by the end of the experimental run, indicating that its flow channel structure is highly susceptible to fouling accumulation and flow impedance. The FCPs of SP-O and SP-2 spacers displayed noticeably smaller increases, and unlike COM, the gradients were linear.

The COM spacer exhibits a nonlinear rise in pressure drop, driven by progressive pore blockage and channel narrowing. Initial fouling partially obstructs certain channels, increasing local flow velocity and shear in the remaining passages whilst slowing flow in the partially obstructed ones. This creates a self-reinforcing cycle where accelerated contaminant transport further constricts certain flow paths, leading to a nonlinear FCP profile. In contrast, SP-O and SP-2 display linear FCP trends due to more uniform fouling deposition. Their optimized geometries promote even shear distribution and reduce localized blockage, effectively decoupling channel constriction from fouling accumulation and enabling stable hydraulic operation.

As shown in Figure 14b, the evolution of permeate flux with time for all three spacers indicates a slowing down of the rate of decline as fouling progresses. With the gradual expansion of the local resistance, the channel pressure drop gradually increases, and the permeate flux decreases. The woven spacer exhibits the lowest initial permeate flux, probably attributable to some screening of the membrane surface. The SP configurations are superior in terms of effective mass transfer.

### 3.5. Energy Consumption

Specific energy consumption (SEC) is the amount of energy that needs to be consumed per unit in the system. Based on the formula of related studies [10,16,46], the following formula is used:

(9) $$SEC=\frac{Q_{p}∆P_{TM}+Q_{f}∆P_{FC}}{Q_{p}}$$

where

$Q_{p}$—Permeate flow rate;
$Q_{f}$—Flow rate of feed;
$∆P_{TM}$—Transmembrane pressure difference;
$∆P_{FC}$—Feed channel pressure difference.

**Figure 15:**
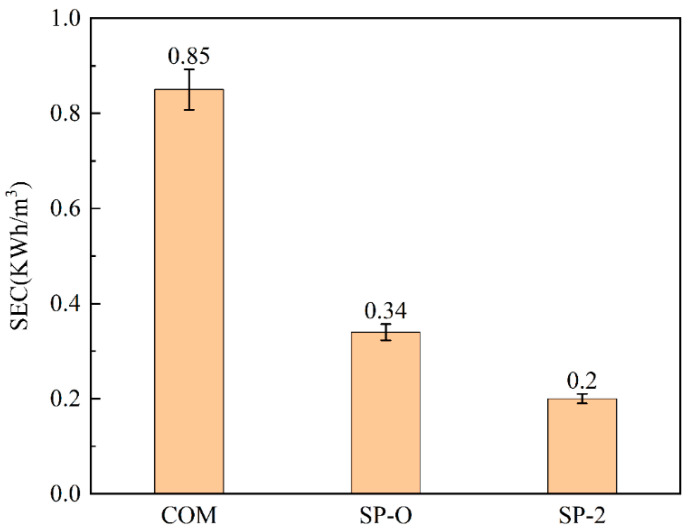
Specific energy consumption of the different feed spacers.

Specific energy consumption serves as a key performance indicator for benchmarking performance, and in the above form, it gives a useful metric for comparing different designs of spacers and modules. Feed spacers simultaneously influence both pressure drop (which influences energy requirement) and the permeate flux (which relates to productivity). For this reason, energy consumption is calculated as specific energy consumption (SEC). These are the relevant terms for a study on a membrane module per se, and it is in accordance with the approach of others [47,48]. In this study, the input flow rate, terminal pressure drop, and constant flux are specified to estimate energy consumption. After 120 h of experimentation, the specific energy consumption was calculated based on the overall amount collected. Figure 15 indicates a specific energy consumption of 0.2 kWh/m^3^ for SP-2, markedly lower than the 0.85 kWh/m^3^ measured for COM. The structures of SP-O and SP-2 are similar, so there is a smaller difference in energy consumption.

### 3.6. Shear Stress and Membrane Fouling

Biofouling performance is a useful way to evaluate a spacer. Most scholars generally agree that biological contamination is related to shear forces. High shear forces positively inhibit biofouling formation, as the biofouling adsorbed on the spacer and membrane surface can be removed at high shear forces. However, a small number of studies have suggested that biofouling is more likely to accumulate at high shear forces, which is related to the concentration and flow rate of the nutrient solution [49,50]. Although the commercial design has a higher average shear, Figure 12 shows that the woven spacer fouled more heavily than the SP spacers. This implies that the elimination of the dead zone for woven spacers is more important than high shear per se. For a given design, higher shear stress is generally beneficial, but the design itself is crucial. Recalling the previous Figure 7, Figure 8 and Figure 9, the SP configurations generate a more uniform flow distribution and low shear. Furthermore, the superiority of these designs was indicated by the simulation output of the DPM model. The coupled CFD-DPM framework enables comprehensive analysis of both fluid dynamics and biocolloid interactions, providing new insights into biofouling mechanisms that were previously unattainable through conventional hydrodynamic studies alone. This represents a significant advancement in membrane fouling research, as it allows for simultaneous evaluation of flow field characteristics and microbial deposition patterns, bridging an important knowledge gap in understanding the complex interplay between hydrodynamic forces and biological adhesion processes in membrane systems.

The DPM results are consistent with experimental observations, suggesting that spacer design may mitigate clogging by altering near-wall hydrodynamics. Specifically, reducing the flow stagnation zone by optimizing filament spacing or surface curvature may disrupt preferential deposition pathways for biofilm. These findings underscore the importance of coupled hydrodynamic and structural optimization in the development of low-fouling modules.

As noted by Beuscher et al. [51] in their paper titled “Membrane research beyond materials science”, the development of a successful process, which has membranes at its core, needs to clear four hurdles. These are listed as a sequence, but “the hurdles … require continuous iteration, backward as well as forward” [28]. The hurdles are (i) material to membrane; (ii) membrane to module; (iii) membrane module to membrane process; and (iv) integration of membrane process into an overall process. The work herein underscores that spacer geometry optimization, rather than membrane modifications alone, should be prioritized for fouling mitigation in systems dominated by spacer-associated biofouling. That is to say that the second hurdle identified in [51] is as important as the first.

### 3.7. Comparative Analysis with the Literature and Practical Considerations

Compared with the aforementioned studies that used wavy [46] or arched structures [10], which were also designed to enhance local mixing, and in that regard, improve performance compared to traditional feed spacers, the SP series spacers achieve a better balance between promoting wall shear stress (for fouling control) and maintaining a lower pressure drop, as demonstrated by our overall FCP trends and specific energy consumption evaluation metrics.

Due to their non-planar wavy structure, the proposed design may use slightly more polymer material per unit length than a standard mesh spacer. However, we believe that this marginal increase in material cost must be assessed in conjunction with the significant operational benefits that include the ability to operate at higher average fluxes without triggering severe fouling issues. As shown in Figure 15, the SP series spacers exhibit lower energy consumption than the COM spacer. Consequently, a lower long-term pressure drop, leading to reduced energy usage, is maintained. Moreover, their superior fouling control capability can extend membrane lifespan and reduce cleaning frequency, thus reducing the environmental footprint.

## 4. Conclusions

This study systematically compares a conventional woven spacer with a novel spacer design that was manufactured in eight configurations. Based upon preliminary CFD and experimental work, two novel feed spacer designs were selected for detailed investigation; these were the spacers SP-O and SP-2, with the latter having a hollow feature. Hydraulic and biofouling performance evaluations of these spacer types and the conventional one (COM) yielded the following key results:

1. The simulation results from Section 3.1 show that the SP spacer type has a more uniform flow pattern distribution than the conventional one, with reduced dead zones and lower FCP.
2. Whilst the channel pressure drop invariably rises with biofouling accumulation for all spacers, the hollow SP-2 maintains a final pressure below 5 mbar, 7.5 times lower than COM and three-quarters the value for SP-O. Simultaneously, permeation flux is proven with SP-2 exhibiting a 23% increase over the COM reference.
3. From the DPM simulation results in Section 3.2, it can be seen that the distribution of biological particles in the SP-X series is sparser than the COM. Analysis using several characterization tools through Section 3.3 shows that the use of SP spacers significantly reduces biofilm deposition on the membrane surface. This was confirmed by further experimental observations; the ATP and TOC content with the SP-2 spacer decreased by 54% and 40%, respectively, compared to COM.
4. The use of SP-2 with its hollow feature significantly reduces filtration energy costs compared to the commercial benchmark, COM. In the laboratory module, the specific energy consumption (SEC) was only 0.2 kWh/m^3^ for SP-2, suggesting a large potential for reducing energy consumption at a commercial scale.

## Figures and Tables

**Table 1:** Geometric parameters of feed spacers.

Type	Length (L mm)	Diameter (D mm)	Width (W mm)	Thickness (H mm)	Angle (°)	Curvature (mm)	Hollow
SP-O	7	0.5	4	1.2	0	1/0.50	NO
SP-1	-	-	3	-	-	-	YES
SP-2	-	-	-	-	-	-	YES
SP-3	-	-	5	-	-	-	YES
SP-4	-	-	-	-	30	-	YES
SP-5	-	-	-	-	45	-	YES
SP-6	-	-	-	-	-	1/0.505	YES
SP-7	-	-	-	-	-	1/0.495	YES

-: Unchanged relative to SP-O.

## Data Availability

The data presented in this study are available on request from the corresponding authors due to privacy concerns.

## Abbreviations

The following abbreviations are used in this manuscript:

ATP	Adenosine triphosphate
CFD	Computational fluid dynamics
DI water	Deionized water
OCT	Optical coherence tomography
CLSM	Confocal laser scanning microscope
FCP	Feed channel pressure drop
SEC	Specific Energy Consumption
SWM	Spiral-wound membrane
TPMS	Triply periodic minimal surface
TOC	Total organic carbon
U_F_	Feed flow velocity (m/s)
k	Boundary layer mass transfer coefficient (m/s)
u	velocity vector of x coordinate (m/s)
v	velocity vector of y coordinate (m/s)
w	velocity vector of z coordinate (m/s)
ρ	liquid density (kg/m^3^)
μ	dynamic viscosity (Pa·s)
P	pressure (Pa)
$m_{p}$	particle mass (kg)
$\vec{u}$	fluid phase velocity (m/s)
${\vec{u}}_{p}$	particle velocity (m/s)
$\vec{F}$	additional force (N)
$m_{p}\frac{\vec{u}-{\vec{u}}_{p}}{\tau_{r}}$	drag force (N)
$\tau_{r}$	droplet/particle relaxation time
$\rho_{p}$	particle density (kg/m^3^)
